# Towards new concepts for a biological neuroscience of consciousness

**DOI:** 10.1007/s11571-020-09658-7

**Published:** 2021-01-26

**Authors:** Camilo Miguel Signorelli, Daniel Meling

**Affiliations:** 1 Department of Computer Science, University of Oxford, Oxford, UK; 2 Cognitive Neuroimaging Unit, INSERM U992, NeuroSpin, Gif-sur-Yvette, France; 3 Center for Brain and Cognition, Universitat Pompeu Fabra, Barcelona, Spain; 4 Department of Psychiatry, Psychotherapy and Psychosomatics, University of Zurich, Zurich, Switzerland

**Keywords:** Autopoiesis, Autonomy, Autobranes, Biobranes, Branes, Consciousness, Compositionality, Models of Consciousness, Metabolic Closure, Organizational Closure

## Abstract

In the search for a sound model of consciousness, we aim at introducing new concepts: closure, compositionality, biobranes and autobranes. This is important to overcome reductionism and to bring life back into the neuroscience of consciousness. Using these definitions, we conjecture that consciousness co-arises with the non-trivial composition of biological closure in the form of biobranes and autobranes: conscious processes generate closed activity at various levels and are, in turn, themselves, supported by biobranes and autobranes. This approach leads to a non-reductionist biological and simultaneously phenomenological theory of conscious experience, giving new perspectives for a science of consciousness. Future works will implement experimental definitions and computational simulations to characterize these dynamical biobranes interacting.

## Introduction

Neuroscience needs new concepts to approach the brain and its cognitive functions (Stern 2017). Many current influential concepts are based on computer metaphors (Daugman 2001; McCulloch and Pitts 1943; Ashby 1957; von Neumann 1958; Wiener 1985; Piccinini 2004; Miłkowski 2018). For example, information processing, integration, codification, and communication reduce the brain complexity to physical computations. These computations leave us without biology, but with new open questions. Some of these questions range from simple to complex, such as how to define brain regions (Stern 2017), how consciousness emerges from physical processes (Chalmers 1995; Nagel 1974), and whether computers might become conscious or not (Dehaene et al. 2017; Signorelli 2018a).

In the field of neuroscience of consciousness that need is evident. For instance, the two most influential theories of consciousness are computational theories (Dehaene et al. 2014; Mashour et al. 2020; Tononi et al. 2016). Their language reduces consciousness to electrochemical neural interactions, without mentioning what is unique in cells and neurons. However, cells and neurons are not only electrochemical, or even more general biophysical mechanisms. Instead, there is something unique in the intrinsic *organization* of cells and neurons which makes them alive. Are these unique and irreducible qualities of life somehow related to the irreducible features of conscious experience?

In this article, we introduce some novel and reintroduce few old concepts to suggest that life is at the core of any sound explanation of consciousness. Instead of treating cells and neurons as performing sophisticated coding and decoding, a better metaphor is the living cell itself: cells and neurons are living beings interacting in order to get food and energy that keep them safe and alive. As such, two neurons do not send or communicate through intricate signals, but may just get and send biological resources. This systemic closure is understood as an operational closure, a more elaborated form of biological autonomy that we will introduce across these pages. We claim that this biological circularity is at the core of the conscious experience, composing a further living closure between multilevel and multidimensional brain-body systems and the animal’s environment.

## Philosophical and experimental perspective

Despite recent progress in the neuroscience of consciousness (Seth 2018), signatures of conscious experience convey isolated experiments about disparate neural correlates (Aru et al. 2012). These different correlates suggest different aspects of the conscious experience, e.g. the phenomenal consciousness and access consciousness (Block 2005), among others (Aru et al. 2012; Bachmann and Hudetz 2014; Tsuchiya et al. 2015; Storm et al. 2017). Unfortunately, these aspects and their neural signatures also lack an integrative explanation (Bachmann and Hudetz 2014; Bayne et al. 2016), as well as a direct link to the phenomenology of consciousness.

We suggest that a sound model of consciousness requires a more promising point of departure: (i) A *radical embodiment reformulation* (Thompson 2004, 2007), and (ii) the integration of brain-body signatures of consciousness in a multilevel organization to reconcile different signatures of conscious experience.

### Radical embodiment

At the core of scientific studies of consciousness lies the hard problem of consciousness. The hard problem of consciousness is a consequence of reducing the mental ontology to the physical ontology. The mental corresponds to unverifiable claims and subjective modes of existence, such as pain or the “redness” experienced only by the subject (subjectivity). Contrary, the physical corresponds to verifiable claims and objects existing independently of others (objectivity). The reduction of the former to the later conveys the question illustrated by Thomas Nagel: “If mental processes are physical processes, then there is something it is like, intrinsically, to undergo certain physical processes. What it is for such a thing to be the case remains a mystery” (Nagel 1974, pp. 445–446). This way of *formulating* the problem implies that “the mental” and “the physical” are two opposed reified substance-ontologies, i.e. two different substances having constant properties and existing each one by itself. On the one side is consciousness (qualia), on the other side, the physical body (with its structure, functions, and mechanisms).

We can avoid this problem by changing our ontologies. Instead of invariant and independent substance-ontologies, we consider variant and interdependent process-ontologies [25]. In this case, the existence is only given by interdependent transformations, and the mental and the physical body become related to each other: they are two different modes of the same existence (Signorelli et al. 2020). Then, in Nagel’s formulation, one can replace the term “physical” by “bodily” and reformulate the above question in the following way (Thompson 2004, 2007): if mental processes are *bodily* processes, then there is something it is like, intrinsically to undergo certain *bodily* processes. In other words, what is it for a physical living body (*Körper/ leiblicher Körper*) to be also a lived body (*Leib/ körperlicher Leid)*)? Critically, the explanatory gap is now between *two types* within *one typology* of embodiment: The living body (*Körper*) and the lived body (*Leib*) are the two modes of appearance of one and the same body. This is called the radical embodiment reformulation.

The radical embodiment reformulation demands two important conditions for a sound model of consciousness: i) consciousness requires a living body, and ii) consciousness cannot be reduced to only neural states. Although the first condition seems evident for any biologist, what makes cells and neurons alive is rarely considered relevant regarding consciousness. The second condition follows the first: because the living body and the lived body (consciousness) are two modes of the same body, it is wrong to assume that the lived body emerges from the living body (including the brain).

Therefore, a sound model of consciousness must account for what makes cells and neurons living entities, including the co-dependence between the living body and the lived conscious body. The radical embodiment reformulation urges us to account for the various biological processes that relate to consciousness without reducing it to neural systems. To tackle these processes, we need to ask: what are the relevant *brain-body* signatures from scientific studies that can inform such a sound model of consciousness?

### Brain-body signatures

Instead of focusing on the necessary and sufficient neural events for conscious experience, we rather ask about the necessary and sufficient kind of *organization* for that conscious experience to occur. In other words, instead of describing one-dimensional interactions only at the level of electrochemical components (cells and neurons), we propose a shift to the relevant interactions at the level of organization (Gershenson 2013a, b; Mazzocchi 2008) *between* various kinds of biophysical components (systems).

To this end, brain-body signatures of conscious experience suggest a multilevel organization, as well as various aspects of conscious experience that need theoretical reconciliation.

One interesting example of brain signatures is the activity of the conscious resting-state brain and its connections with brain-body activity. At resting state, studies of functional magnetic resonance imaging (fMRI) show that brains present intricate anticorrelated activity (Biswal et al. 1995; Fransson 2006; Fox et al. 2005; Pessoa 2014). In many cases, this activity is simulated by dynamical systems at the edge of criticality (Deco et al. 2008; Breakspear 2017). These models need to adjust different parameters, among those, noise plays an important role (Ghosh et al. 2008; Deco et al. 2009). Noise is associated with the intrinsic noisy cellular and neural activity (Faisal et al. 2008), and it also relates to the physiological coupling between the brain and the rest of the body as nonstationarities reveal (Thompson and Varela 2001; Laumann et al. 2017; Nguyen et al. 2016). The existence of these nonstationarities suggests that brain and body systems are interconnected. Two examples are the interconnected brain-stem system, which regulates homeostasis, and nuclei that regulate sleep and wakefulness (Thompson and Varela 2001). These couplings change during sleep (Bashan et al. 2012; Bartsch et al. 2015; Ivanov et al. 2017) and under different anaesthetics (Stankovski et al. 2016). Even more fascinating, part of this bodily activity seems to influence conscious perception (Park et al. 2014) and confidence (Allen et al. 2016).

The molecular environment and metabolism also regulate these brain-body couplings (Haydon and Carmignoto 2006; Petit and Magistretti 2016; Jha and Morrison 2018). In relation to conscious activity, evidence suggest that glial cells and their energetic production is involved on these regulations (Bélanger et al. 2011; Ramadasan-Nair et al. 2019; Perouansky et al. 2019; Velazquez 2020). Another example corresponds to the minimal energetic requirement which is necessary to recover consciousness from chronic impairments (Shulman et al. 2009; Stender et al. 2016; Di Perri et al. 2016). It implies a relevant metabolic coupling. Furthermore, if one compares disorders of consciousness with normal awake subjects, dynamical changes are observed in the form of a reduction of the brain repertoire (Demertzi et al. 2019). This intricate dynamics of healthy brains is partially recovered using deep brain stimulation in different zones of the chronic impaired brain (Schiff 2010, 2013; Koubeissi et al. 2014; Corazzol et al. 2017). Consciousness loss during applications of anaesthetics also show similar dynamical signatures (Barttfeld et al. 2015; Uhrig et al. 2018), but differently, anaesthesia presents two types of emergence modes from sedation. One of them is a very graded and gradual emergence, whereas the other generate abrupt arousal, typically followed by disorientation and sudden movements (Canet et al. 2003; Lepousé et al. 2006). Under anaesthesia, the induction and emergence also present asymmetries (Lee et al. 2011; Chander et al. 2014; Warnaby et al. 2017). All together, remind us about the importance of biochemical and molecular interactions, mainly between endocrine, immune systems and neural systems (Thompson and Varela 2001).

Finally, these brain-body activities generate different brain signatures of consciousness associated with different aspects of consciousness. For example, during sleep states, electroencephalogram (EEG) activity and body rhythms show clear physiological changes and transitions (Simon and Emmons 1956; Brown et al. 2012). Unlike anaesthesia, those changes are natural and gradual. During dreams states, EEG measurements reveal brain activity mostly in the parietal-occipital cortex (Siclari et al. 2017). It is associated with phenomenal consciousness. Contrary, in awake conditions, experiments on conscious perception in humans and other primates, convey evidence about a frontoparietal-cingulate network and ignition activity from the frontal cortex to the rest of the brain (Van Vugt et al. 2018). It is called access consciousness (Block 2005). In this mode, the trajectories of brain states seem to accelerate when someone perceives a stimulus, compared with the opposite situation (Baria et al. 2017). It suggests transient dynamics of access consciousness. Other distinctions indicate two different cognitive systems (Shea and Frith 2016; Herzog et al. 2016; Dehaene et al. 2017; Signorelli 2018), associated with two conscious processes: the awareness of content (awareness) and the awareness of the processing on these contents (self-reference or self-monitoring).

Are these signatures and distinctions conflicting evidence about the neural correlates of consciousness? (Boly et al. 2017). Taking them in isolation, probably yes. However, taking them as a whole, these signatures and modes of consciousness may correspond to different brain-body couplings and dynamical phase transitions.

The evidence above supports a multilevel organization, where the molecular environment, cellular organization and neural systems interact to ensure conscious experience (Thompson and Varela 2001; Prentner 2017; Kringelbach et al. 2020). These multilevel cycles and processes between brain and body underpin the integrity of the organism as a whole (Thompson and Varela 2001). The body activity relevant to brain activity may define a subjective frame supporting subsequent conscious experiences through interactions of neural and cellular responses (Velazquez 2020) to external but also visceral stimuli (Critchley et al. 2004; Seth 2013; Park and Tallon-Baudry 2014). The body activity signalled to the brain, and this subjective frame may represent different brain-body systems interacting. The various transitions, asymmetries observed during sleep and anaesthesia, and diverse network signatures of phenomenal consciousness and access consciousness, may reflect the degrees of couplings of these different systems and the dynamical phase transitions triggered by them (Werner 2012, 2013).

As a consequence of this discussion, we propose that a sound biological model of consciousness must integrate conscious experience in its irreducibility, in order to constitute a comprehensive framework.

## Concepts for a biological model of consciousness

According to the previous discussions, the aforementioned conditions impose further requirements. On the one hand, the definition of the living body needs to capture the uniqueness of living beings in contrast to non-living things. On the other hand, we need a principle to explain the mutual relationship between the living body and the lived body, i.e. their co-dependence at different scales.

In the following, our framework provides a concrete implementation and extends the original embodiment conjecture (Merleau-Ponty 2005; Thompson and Varela 2001): consciousness relies on how brain dynamics are embedded in the somatic and environmental context of the animal’s life.

### Closure and biological autonomy

**Fig. 1:**
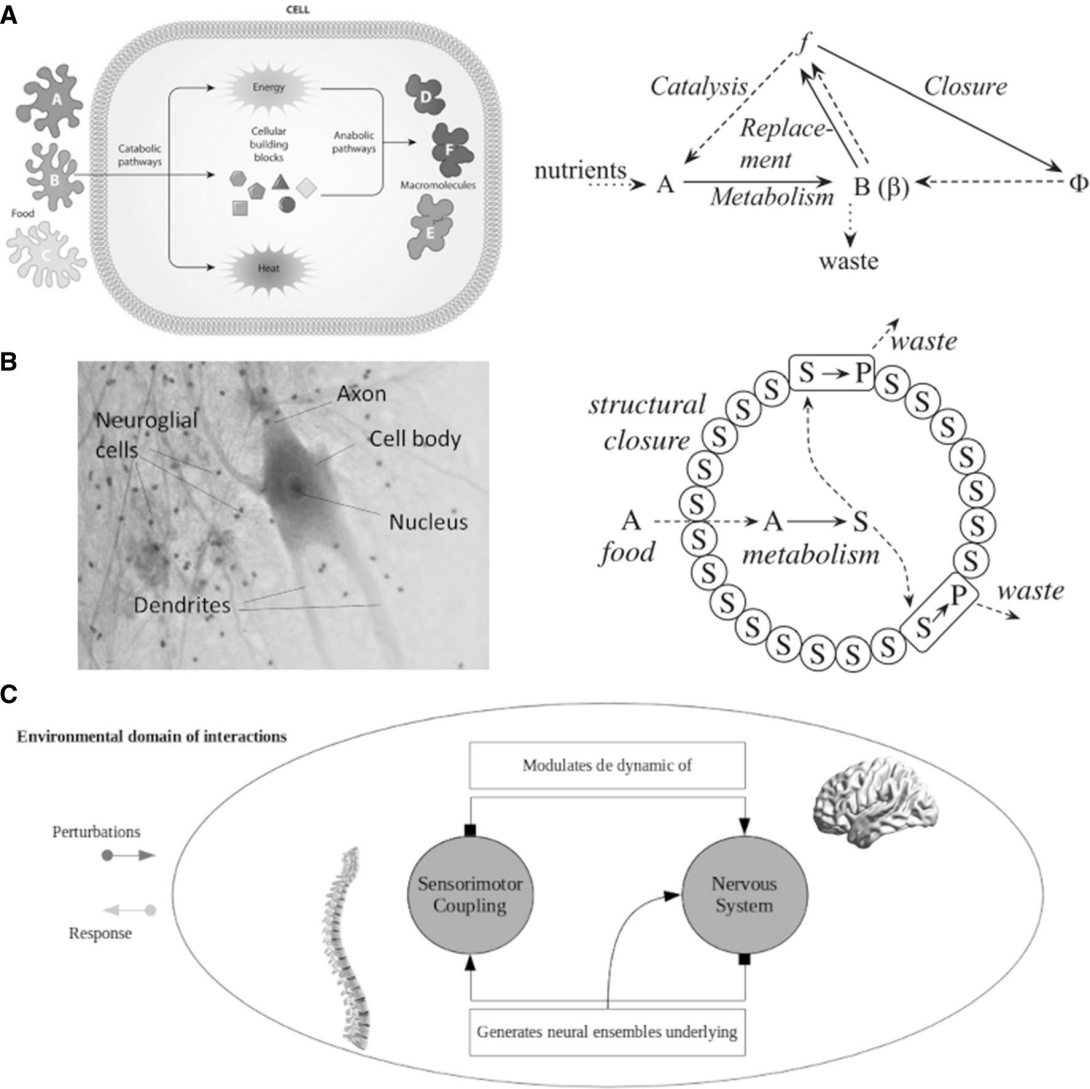
Different types of closure. **a** Metabolic closure refers to the assumption that “all catalysts needed for metabolism are themselves products of metabolism” (Cárdenas et al. 2010). Cellular metabolism corresponds to the set of chemical reactions to maintain cellular life. The left diagram summarizes one way to represent the closure of these reactions. Dashed arrows represent catalysis, and continuous arrows represent transformations of matter by chemical reactions (Letelier et al. 2011). Metabolism is a set of chemical transformations $A\rightarrow B$, catalysed by enzymes *f*. Replacement corresponds to the re-synthesis of *f* by a replacement system $\phi$. Enzymes are synthesized from the products of metabolism, requiring other catalysts so that $\phi \rightarrow B \rightarrow f$. Then, closure becomes the continuous replacement of any catalyst, such that the diagram is closed (Letelier et al. 2011). Figures adapted from O’Connor and Adams (2010) and Letelier et al. (2011). **b** Living systems are also structurally closed. This applies to cells, neurons and glias. The concept of autopoiesis signifies this closure as described by the left diagram. In that diagram, dashed arrows are physical movement while solid arrows represent chemical reactions. In this sense, autopoietic systems become “encapsulated systems” (Letelier et al. 2011). The metabolic reactive network produces molecular components that determine the bounded system that generates the metabolic reactive network (Thompson 2007). Diagram adapted from (Letelier et al. 2011). **c** The nervous system is one example of biological autonomy and organizational closure. In this case, the closure is at the level of patterns of activity. Sensorimotor coupling modulates the nervous system that reciprocally generates patterns of activity shaping the sensorimotor system. In other words, the nervous system is immersed in a loop of activity where sensory input defines motor output and vice versa. Diagram adapted from Thompson (2007)

The starting point of our model is the living system. One way to distinguish living systems, such as cells, neurons, and bacteria, from non-living systems, draws on a living system’s distinct network of internal productions (Varela et al. 1974; Maturana and Varela 1998; Ruiz-Mirazo and Moreno 2004; Maturana 2011). In theoretical biology, this internal cellular organization is referred to as closure (Varela et al. 1974; Letelier et al. 2006, 2011; Cárdenas et al. 2010). There are, however, different notions of this closure, and one form to refer to them is called metabolic closure (Fig. 1a). Metabolic closure means that all the catalysts needed to stay alive are produced by the organism (Letelier et al. 2011): “molecules that define the metabolic network of a cell, whether metabolites or enzymes, are produced by processes which are themselves mediated by other molecules produced by the very same metabolic network”. Those biochemical reactions constitute metabolism from which enzymes and other proteins participate in those reactions as well as are the product (metabolites) of those reactions (Letelier et al. 2011). One example of a metabolic reaction is the glucose metabolism and its different profiles in neurons and astrocytes (Magistretti and Allaman 2015). In the case of the glucose metabolism the reaction is catalysed by the enzyme glucokinase: $Glucose + ATP {\mathop\rightarrow\limits^{G}} Glucose-6-phosphate + ADP.$ This reaction can be seen as the action of an operator *G* transforming the input molecules into the output molecules. The internal set of participating molecules, enzymes and proteins signify the closure: Sometimes they operate as catalysers and at other times as inputs or outputs (Fig. 1a). Hence, the organism becomes distinct from its environment through its dynamics of production. The product of this metabolic process of production is the producer itself. In other words, living systems exhibit a particular form of *closure* (see Letelier et al. 2011 for a detailed discussion).

#### Definition 1

Living systems exhibit closure: They are sustained as a network of processes that are recursively dependent on each other.

Closure makes cells and neurons unique. One form to specify that closure corresponds to the concept of *autopoiesis* as structural closure (Cárdenas et al. 2010). An autopoietic system is itself a network of biological recursive and intertwined actions of components and production (Varela et al. 1974; Maturana and Varela 1998; Ruiz-Mirazo and Moreno 2004; Maturana 2011). These biological actions imply a context of spatial-topological boundaries or “membranes” (Fig. 1b), which also require and define the component production network (Ruiz-Mirazo and Moreno 2004; Thompson 2007). These interactions continuously regenerate their own network of processes and enact a concrete topological unit in space (Varela et al. 1974). Therefore, cells and neurons are closed systems: Instead of being static entities, they only exist as arising and temporally sustained networks of recursively interacting processes.

#### Definition 2

Biological closed systems internally produce what then constitutes their operation of production.

Autopoiesis is considered one of the possible minimal set of requirements to define what is living and what is not (Varela et al. 1974; Letelier et al. 2011). It conveys three minimal criteria that any autopoietic organization needs to satisfy: a) Semipermeable boundary: Does the system have a boundary that allows us to distinguish between inside and outside in relation to its relevant components?; b) Reaction network: Are the components being produced by a network of reactions inside the boundary?; c) Interdependency: Are conditions a) and b) interdependent? Are the components of the boundary being produced by the internal network of reactions as well as this network is regenerated by conditions from the boundary itself? If a system meets these three criteria, then the system is an autopoietic organization (Table 1).

**Table 1:** Autopoietic systems according to three classification criteria. Table from Thompson (2007)

**Entity**	Boundary	Network	Interdependent	Is autopoietic?
Virus	Yes	No	No	No
Crystal	Yes	No	No	No
Bacterium	Yes	Yes	Yes	Yes
Amoeba	Yes	Yes	Yes	Yes
Mitochondria	Yes	Yes	No	No
DNA section	No	No	No	No
Autocatalytic set	No	Yes	No	No

The paradigm example for autopoiesis is the living cell. In a living cell, the constitutive processes are chemical. Those chemical metabolic reactions recursively depend on each other. This means that, in order to occur, one chemical metabolic reaction requires the products of other chemical metabolic reactions. Those reactions mutually depend on each other. By this, their whole network of relations constitutes the living cell as a unity in the biochemical domain. Interestingly, this constitution of the living cell as a unity takes a special form as spatial boundary (Thompson 2007). This spatial boundary is realized through the living cell’s membrane that enables metabolic reactions while the metabolic reactions bring forth the cell’s membrane. This mutual dependence is at the core of autopoiesis (Letelier et al. 2011).

Autopoietic systems are a specific kind of *autonomous systems*. As living systems are metabolically and structurally closed, they are open systems in terms of thermodynamics. In other words, they are connected with the environment to obtain the energy that its metabolism requires. In Fig. 1a, b, this is represented by nutrients, food and the irreversible reaction of producing waste. However, external causes do not modify the internal organization but may contribute or modulate reactions to them (Fig. 1c). The inputs and outputs of biologically closed organizations come from and go to the environment (Thompson 2007). The closure property is not about the exchange of energy or materials, it is about how this exchange is regulated. In a biological system the flow of energy that keeps the system away from the thermodynamic equilibrium is regulated by the organization of the system itself (endogenous self-organization), while in the case of a physical system, it is controlled by external mechanisms. The first condition defines an autonomous system, whereas the second condition defines a heteronomous system (Thompson 2007). The former develops internal, local, and global processes to stay away from thermodynamic equilibrium (Ruiz-Mirazo and Moreno 2004), keeping its intrinsic dynamic, while the latter is determined by external mechanisms (Thompson 2007).

#### Definition 3

Biological autonomy is the closure between the internal productions (metabolism) and the external extensions of this internal organization as actions in the environment (agency) (Ruiz-Mirazo and Moreno 2004).

Biological autonomy makes use of a more general instance of closure. In this case, closed interactions define only virtual boundaries (nonphysical/non-material membranes), i.e the closure is operational (Varela 1979, 1997). In other words, what is now being regenerated is the internal topology, not the components. This is called *operational closure*, where all the dynamic processes to keep the organization of the system are maintained or sustained by the system: They construct and reproduce their own internal topology. Examples of such systems are microbial communities, immune system, the nervous system, neural assemblies, multi-cellular organisms, but also insect colony, or animal society, among others.

In contrast to the aforementioned autopoiesis, the realization of operational closure in, for example, multi-cellular organisms and neural assemblies does not involve a *spatial* boundary. Rather, they bring forth an *identity* constituted through the recursive network of relational processes without a fixed physical membrane. In the case of an autonomous social network such as an insect colony, the boundary is social and territorial, not material. While metabolic or autopoietic closure enacts a minimal bodily unity at the metabolic level, another example, the sensorimotor closure as in the case of the nervous system (Fig. 1c), brings forth a sensorimotor unity at the perception-action level (Thompson 2007; Varela 1997).

In summary, we characterized a living system by its metabolic and operational closure. This closure presupposes the notion of biological autonomy: “[e]very autonomous system is organizationally closed” (Varela 1979, p. 58), i.e. operationally closed^1^. This makes closure and autonomy deeply interdependent concepts which emphasize the system’s dependence on a network of recursively interacting processes.

### Compositionality and co-arising

If the relationship between the living body and lived body is co-dependent, how does this co-dependence work? Dynamically, one can understand this co-dependence as local *bodily* processes giving rise to novel global *consciousness* processes that have “their own features, lifetimes, and domains of interaction” (Thompson and Varela 2001 p. 419). Simultaneously, those global characteristics of a system’s conscious activity constrain the local interactions on the body level (Thompson and Varela 2001; Rodríguez 2008). In other words, none of the two is reduced to the respective other, they co-arise.

#### Definition 4

The co-arising of the living body and the lived body implies that they reciprocally depend on each other. None of the two can be reduced to the respective other.

In our framework, co-arising becomes a principle of *compositionality*: the parts and the whole are mutually defined. In Evan Thompson’s words, “part and whole are completely interdependent: an emergent whole is produced by a continuous interaction of its parts, but these parts cannot be characterized independently from the whole” (Thompson 2004, p. 391). This interdependence of parts and whole is called *dynamic co-emergence*. It reflects the notion that (i) the parts give rise to the whole, (ii) the whole gives rise to the parts, and that (iii) none of the two can be reduced to the respective other, they co-emerge. Therefore, dynamic co-emergence refers to the idea that both propositions apply simultaneously. Following the examples above, the inside (“sensorimotor self”) and the outside (“environment of otherness”) co-emerge through nervous system’s operational closure (sensorimotor closure) at the level of perception and action (context). This notion of dynamic co-emergence shows important parallels to the idea of *compositionality*.

**Fig. 2:**
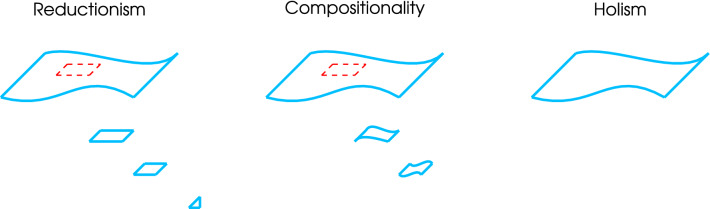
Compositionality. Take the example of oscillatory membranes. In order to analyze them, reductionism will divide the membrane into small pieces (e.g. lipids, proteins, ion channels, etc). In the process, the global properties of the membrane are usually lost, in this case the whole oscillation. In general, a reductive approach allow us to understand the physical components, but it is unable to recover the whole (usually emergent properties) by the mere description of the parts. Contrary, a compositional approach divides the membrane considering the property of the whole being described. In this example, its capability to oscillate. The smallest component is indeed a group of oscillators. As such, the whole property is always present in the relationship of their parts, and the composition of their parts is much more than the sum of them. Finally, a holistic approach would negate the possibility to explain the membrane oscillation by the mere sum of their parts. Like compositionality, holism claim that the parts of a whole and the whole are interdependent, but differently than compositional approaches, these parts cannot exist without the whole

Here, compositionality formalizes this interdependent relationship. A new whole is a composition of its parts only if the whole has the properties of its parts and vice versa (Fig. 2). In other words, the system is non-trivially decomposable (Coecke 2011; Coecke et al. 2016). Importantly, compositionality is not the same as composing. Composition highlights the parts, while categorical readings of compositionality accentuate the whole, such that the parts need to be defined by the properties we want to describe in the whole. In other words, any compositional division is contextual to the whole property described (Atmanspacher and Rotter 2008). For example, if we want to recover whole-brain oscillatory activity, the minimal section in our system analysis becomes a group of oscillators interacting. This partition is independent of the physical partition of the brain organization, i.e. it is operational: we divide the brain according to the operation we want to describe. In category theory, a branch of mathematics, the composition of two morphisms (processes) $f:x \rightarrow y$ and $g:y \rightarrow z$ in a category, needs to produce another morphism, such as $g \circ f:x \rightarrow z$. The new morphism $g \circ f$ is called the composite of *f* and *g*. Compositionality forces us to define the parts and the whole simultaneously, demanding a principle which is neither reductionism nor holism (Fig. 2).

#### Definition 5

The whole is constituted by the relations of the parts, and the parts are constituted by the relations they bear to one another in the whole (Thompson 2004).

The radical embodiment also implies a contextual relationship between the whole and parts. The output of any biological experiment will depend on the feature observed in the biological system, but the same observed system, regarding a different context, might bring other conclusions. In the neuroscience of consciousness, this contextual relationship appears on the multiple neural signatures of consciousness. Different experimental conditions point out to different neural correlates of consciousness (Aru et al. 2012), e.g. prefrontal-parietal networks are relevant during conscious perception (Van Vugt et al. 2018), but posterior cortical regions are dominant under conscious dreams (Siclari et al. 2017). According to the embodiment conjecture, we cannot escape to this contextual behaviour of neural activity, because this activity is immersed in a body with complex intertwined relationships. As a consequence, the definition of the relevant brain-body organizations for conscious experience is compositional and contextual to the whole experience.

### Biobranes and autobranes

Biology is all about autonomy, and biological membranes seem to signify this autonomy. Biological membranes play the role of boundaries between the living system and the environment. They regulate the exchange of resources, protect the internal system, among other important functions. It is not surprising that the core idea behind the concepts of autopoiesis and metabolic closure is indeed a formalization of the intuitive notion of biological membranes. The external membrane in cells is not just part of the network of internal production that then become elements of the membrane itself, but it is a topological closed system: there is no starting nor ending point in the cell membrane. Then, operational closure becomes a generalization of biological membranes aiming to incorporate virtual systemic boundaries, such as the non-material boundaries that define the intertwined social relationships in a group of animals. In this case, the boundary is organizational.

In physics, an acronym for membrane is *brane*. A brane is a *n*-multidimensional dynamic object that posses energy in form of tension over its volume. This energy becomes the energetic source for certain interactions, while the observable universe comprises the internal volume of that brane. Mathematically, the dynamical evolution of a brane is a map $\varphi :W \rightarrow M$, where *W* is a reference manifold with n+1 dimension and *M* represents the “spacetime” through the brane propagates (Moore 2005). In this nomenclature, $\varphi (W)$ is called the *worldvolume*. Moreover, branes wiggle and bend through oscillations. These oscillations are sections of the normal sheaf to a subset $\varphi (W) \subset M$. Then, different mathematical structures^2^ are added to *W* and *M*, in order to study different phenomena. One example of such branes is the surface of the ocean (Moore 2005), while the best-known example of branes is associated with string theory and theories of gravity (D-branes). In this last example, a brane corresponds to local boundary conditions preserving multidimensional invariance (formally, conformal invariance) and the tension *T* becomes a key feature to define different types of particles and cosmological scenarios.

The concept of brane is relevant to our discussions because we can extend and formalize our biological intuitions without the need of reducing the brain to mere computations. Apart from the mathematical structure that defines branes, what also makes branes different than other theoretical descriptions, such as dynamical systems theory, is their capability to recover other physical systems, i.e. topological branes may become primary structure. For example, dynamical systems describe organizations usually evolving in time, and therefore making time the independent variable. In this case, the group of independent variables take the role of a fundamental dimension of description in which the system evolves. In the case of branes, they might be treated as physical self-sustained systems, i.e. their energetic interactions depend only on the brane volume. As such, the intrinsic structure is what defines the worldvolume, and we can further interpret this worldvolume in the context of biological entities. In this context, an interesting conceptualization is the D-branes as primary or the fundamental organization from which spacetime and other dimensions emerge. As such, spacetime might arise from purely topological branes. In biology and cognition, these brane-structures may represent the different brain-body organizations that give rise to the cognitive space and time from mutual constraints between the environment and the biological agent (Signorelli et al. 2020). In other words, the biological space and time becomes embodied. Therefore, instead of reducing our biological membranes to physical branes, we conjecture that biological branes related to conscious experience would be as fundamental as the fundamental branes in theoretical physics.

With this conjecture in mind, we now introduce an extension of biological autonomy: the concept of *biobrane*. Closure and biological autonomy lead to organizational invariant (Letelier et al. 2006), self-organized, and self-regulated systems (the other way around does not always apply). These systems are called biological autonomous systems and their organizational invariant may take the form of either concrete membranes (e.g. cells and neurons) or virtual boundaries (e.g. immune system). Thus, we define a biobrane as all the possible closed biological membranes/boundaries at the meso-scale of a whole biological organism that self-sustain their interactions in relationship with conscious experience.

#### Definition 6

A biobrane is a multidimensional dynamical description of biological autonomous system forming a unity (operational closure and self-regulation), in the form of concrete or virtual meso-scale membranes of an organism.

Using compositionality, a particular biobrane is then the autobrane. If a biobrane is both operationally closed and composed of units with metabolic closure (specially autopoietic units), they are called autobranes and entail a double closure composition.

#### Definition 7

An autobrane is a biobrane operationally closed and self-regulated, composed by elements that are operationally closed and self-regulated as well.

We propose that biobranes and autobranes are a more powerful conceptual framework and its mathematical machinery may describe the notions of closure better than dynamical systems.

Biobranes and autobranes are extensions of biological networks, cells and neurons, such as D-branes are generalizations of close and open strings. This formal analogy is, however, just an analogy. We do not claim that biobranes are built in the same way than cosmological branes, but that their mathematical structure is similar^3^. In other words, we can model biobranes using the mathematical machinery of branes, up to certain distinctions (e.g. we might not need quantum branes at the Plank scale). This approach follows the common pragmatic use of the same type of differential equations to model an endless number of different physical and biological phenomena. For example, the *cable equation* is a useful equation modelling the propagation of electromagnetic signals in a cable, as well as a useful approximation of the propagation of action potential in pyramidal neurons.

In this context, the radical embodiment and its connection with dynamical system theory is more practical than essential (Thompson 2007). This link looks for a mathematical formalization of the biological autonomy in neural systems that “actively generates and maintains its own coherent and meaningful patterns of activity, according to its operation as a circular and reentrant network of interacting neurons” (Thompson 2007). Biobranes and autobranes may provide that formalization. In our case, the biological closure discussed above might be modelled by the different topologies and biobrane volume, its *T* tension as intrinsic biological energy and its worldline as the dynamical evolution. These applications are left for future works, while we focus here in its conceptual introduction.

An important remark is that modelling does not imply a reductive or ontological metaphor. The metaphor to conceptualize these biobranes as a living organization is to understand their interactions in a similar way that two independent living beings interact, e.g. two amoebas. We understand that “our organism is a meshwork of “selfless selves,” and we are and live this meshwork” (Varela 1991). Therefore, biobranes would act and behave as independent functional organisms, while autobranes as functional and anatomical organic units.

**Fig. 3:**
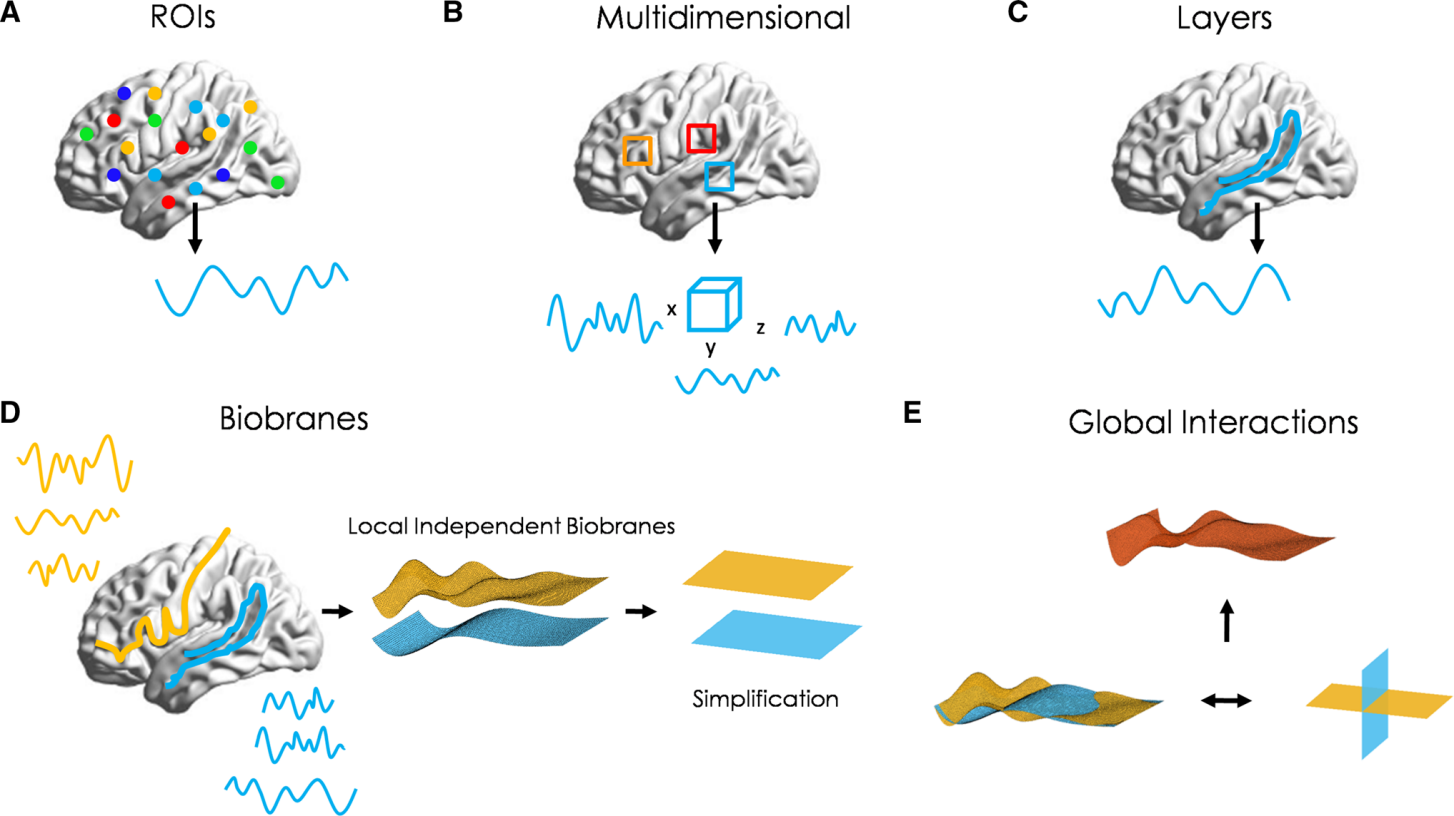
Biobranes. **a** In neuroscience, the brain parcellation across regions of interest (ROIs) is a common assumption. These regions form anatomical or functional brain networks, and its physiological activity is normally averaged to get one single time-series activity per node (light blue line). **b** Recent brain parcellation techniques define multidimensional time-series, using principal component analyses or other multivariate methods. The goal is to describe more complex multidimensional connections between brain regions. **c** Additional efforts focus on the physiological activity of different spatially extended regions, mainly cortical layers. In this case the anatomy and function define a whole-unite (the layer). **d** Our discussion extends previous assumptions to a brain-body organization, where each colour corresponds to one family of multidimensional biobranes. For visualization purposes, we plot two toy examples of hypothetical three dimensional branes. Each dimension may correspond to physiological signals (functional activity), like in multidimensional approaches, as well as kinetic changes of the whole (anatomy), metabolic exchanges, among others. In the general case, biobranes are not restricted to three or four dimension. To reason about the connectivity between biobranes, we can represent them as colour layers, a visual simplification. **e** By definition, these biobranes are independent organizations under unconscious conditions. Then, the interaction among biobranes generates new dynamical conditions due to breaks of symmetries within the biobranes. This is visualized by the overlap of their activities (bottom right) or by rotation layers (bottom left). This overlap composes a new “many colours” biobrane (top)

Furthermore, biobranes and autobranes may generalize previous neurophysiological divisions of the brain anatomy and its function. For example, the simplest way to analyse the brain is to parcel it in regions of interest (ROIs) and average the physiological activity in each of these regions (Fig. 3a). More recent efforts are focusing on multidimensional activity (Fig. 3b). In this case, the brain region is not reduced to one dimension of physiological activity but becomes a three dimensional or bigger dimensional object (Basti et al. 2020). Dimension are usually taken from principal components analyses of electrophysiological signals (EEG, fMRI, among others). Other attempts define extended anatomical and functional regions as the minimal unit of analysis, mainly cortical layers (Fig. 3c). This is called layer-approach and focus on layer-fMRI analyses (Huber et al. 2020). In this line, autobranes correspond to multidimensional layers with structural boundaries, while biobranes are multidimensional layers that not only incorporate neural systems, but also more general brain-body systems. In figure 3d we give a hypothetical example. The anatomical and functional parcellation is translated to virtual multidimensional membranes as abstractions of biological autonomous systems. The dimensions of biobrane activity include electrophysiological, metabolic, kinetic, among any other relevant physiological activity that characterizes, ensure the unity and the survival of the biobrane.

Biological realizations of biobranes may range from some cortical layers in the brain to the immune system in the body. The most relevant autobranes might convey neural layers and cell-glia layers (Velazquez 2020).

In short, biobranes and autobranes are new and relevant concepts for brain functioning in the context of conscious experience. First, the conceptual introduction of topological biobranes may lead to the mathematization of operational closure. From that, other closure compositions can be described. Secondly, we can implement multidimensional physiological brain-body signals using multidimensional approaches in biobranes. These dimensions may include anatomical, functional and metabolic interactions, among others. Thirdly, we can treat spatial and temporal dimensions as embodied in the biological agent, instead of treating them as independent variables external to the agent. In other words, physical space-time is no longer the theatre in which experience appears, but cognitive space-time might arise from experience, as biobranes and autobranes interact. Finally, biobranes and their ability to oscillate in multiple dimensions may serve to explore co-dependent configurations of conscious experience and brain-body signals, as they trigger each other. We discuss this final approach in the next section.

## Closed biobranes composition

Following the main definitions above, we now introduce the main hypotheses regarding brain functioning and consciousness interaction.

### Brain-body architecture

Our first hypothesis generalizes the brain division to a membrane division of the brain and the body. In short, we propose a biobrane structure and *multibrane structure* (Fig. 3d, e). Please notice that the common practice of region parcellation in cognitive neuroscience is, in fact, a weak form of our postulate:

#### Proposition 1

*The brain and the body allow for a multibrane structure.*

The multibrane structure is a group of multiple dynamical biobranes, here represented by layers. Each layer stands for an independent biobrane with its particular type of internal interaction and/or components. The brain multilevel structure now becomes a multidimensional group of biobranes interacting.

A biobrane of neurons may be defined by their main interactions through action potentials, while a molecular biobrane may interact by chemical gradients. These internal interactions represent local interactions across the biobrane. Then, interactions between biobranes correspond to branes acting as bridges between other branes and illustrated by rotation layers (Fig. 3e). These types of interactions become global interactions and break the local dynamic, generating new dynamics and symmetries in the more general multibrane structure (Fig. 3e). For example, a neural biobrane interacting with a molecular biobrane might make available more neurotransmitters to the neural biobrane, changing the neural biobrane dynamic, as well as this new activity changes the concentration of these chemicals and the dynamics of the molecular biobrane. The sustained interaction interferes with the biological stability of the original branes, triggering different responses in each other: a) breaking observed default synchrony as local integration across regions inside biobranes and b) spreading new activity through them.

An important observation is that the brane rotation is not material (Fig. 3e), i.e. regions in the brain-body do not rotate as their mathematical representations. This point is evident in three-dimensional models of aggression (fear, behaviour and rage dimensions), where the animal in question is not physically moving in that topological manifold. The scientist models the mood of the animal (Zeeman 1976). In our case, rotations of layers are graphical characterizations of the membrane oscillatory activity: With increasing activity, overlaps with other biobranes increase, and therefore new influences in their intrinsic dynamic come into play.

### Compositional consciousness

We hypotetize that, poetically speaking, conscious experience is the biological universe of cosmological constellations supported by biobranes and autobranes interacting. Conscious experience is co-defined by the close coupling and compositional interactions between metabolism, autonomy and the animal’s environment such that any conscious action returns to the animal in a meaningful way to that animal.

To understand this idea, we first define compositionality for brane structures. The inter-brane interactions may become compositional, only if the new global system compounded by the branes is also a brane, i.e. the new system is also closed and self-regulated.

#### Proposition 2

*If two or more branes and their interactions generate a new global system of branes which is operationally closed and self-regulated, it is a composition of the former branes.*

These brane compositions extend to biobranes and autobrane compositions. Autobranes compound other autobranes as long as the properties of autobranes, i.e. closure of the system and closure of their unites, still hold.

Following previous discussions, life is characterized by the closure of intertwined component productions, while biological autonomy is signified by the operational or organizational closure of these components (Table 2). In the embodiment framework, a cognitive agent corresponds to the coupling between the agent and the environment so that recurrent sensorimotor patterns of perception and action appear (Thompson 2007) (Fig. 1c). These patterns modulate but do not determine the endogenous activity, while the endogenous activity informs sensorimotor coupling. The internal realm is not a representation of the external. However, their mutual relationship is enacted by the living agent and the coupling mode with the environment. This recursive action, the closure of living systems and its environment, creates meaning and involves a *minimal lived experience*. This is the sense-making dimension that becomes the “intentionality in its minimal and original biological form” (Thompson 2004; Varela 1997).

**Table 2:** Closure composition and co-arising of conscious experience

Kind of closure	Characterize	Supporting process	Description
Compositional closure	Conscious experience	Multibrane	Closure among different levels of closure
Operational closure	Identity	Biobranes (biological autonomous systems)	Closure of the internal topological organization
Structural closure	Life	Autopoietic organization	Closure of component production

We can extend these intuitions and claim that different living organizations convey different types of such experiences, and one particular form of these experiences is what we call *conscious experience*. In other words, we propose that consciousness is a non-trivial composition at different levels of the biological closure defined above. Therefore, the organization of consciousness requires the organization of life. It responds to the increasingly influential view that crucial processes for consciousness cut across brain-body–world divisions, rather than being mere brain-bound neural events (cf. Thompson and Varela 2001).

The lived experience is to be seen as irreducible, since any perception of a world is enacted *co-dependently* through the system’s biological organization (Thompson 2007): Our biological organization shapes the world we experience. In this context, conscious experience becomes a composition of living experiences, such that they are mutually defined and form two modes of the same process of closure.

Conscious experience entails a close loop of brain functional activity (Llinás and Paré 1991; Llinás 2003), but also a closure at the metabolic and structural level, creating a sense of the interaction in question (Thompson 2007). The experience becomes a conscious experience when the activity of the system returns as meaningful benefits to the whole system, i.e. the system not only enacts the environment (autonomy) but projects “intuitions” that in a short temporal scale reward the internal organization and at long term convey future additional benefits to the system.

The whole multibrane and the parts are mutually defined. In other words, the operational and metabolic closure is partially inherited from their components, but importantly, although the closure is the same operation, the objects of that closure are different. The closure of cells and neurons is at the level of molecular components (self-production of components), while the closure of biobranes and autobranes is at the level of dynamical organization (systems that self-reproduce their organizational complexity). The composition between these two closures generates a biological autonomous system which is both operationally closed and structurally coupled with its environment (Thompson 2007). The new compounded system is autonomous in a new form. The whole multibrane system compounded by autonomous biobranes and autobranes, self-sustains and self-produces its structural and organizational dynamic through closed interactions at various levels.

### Aspects of consciousness

We suggest that these special types of compositions, illustrated via compositional interactions of autobranes and biobranes, are involved in the co-arising of different aspects of conscious experience as a new closed system: Conscious experience co-arises with global interactions of biobranes and autobranes.

#### Proposition 3

*Compositional interactions of autobranes, biobranes and branes co-arise with aspects of conscious experience.*

**Fig. 4:**
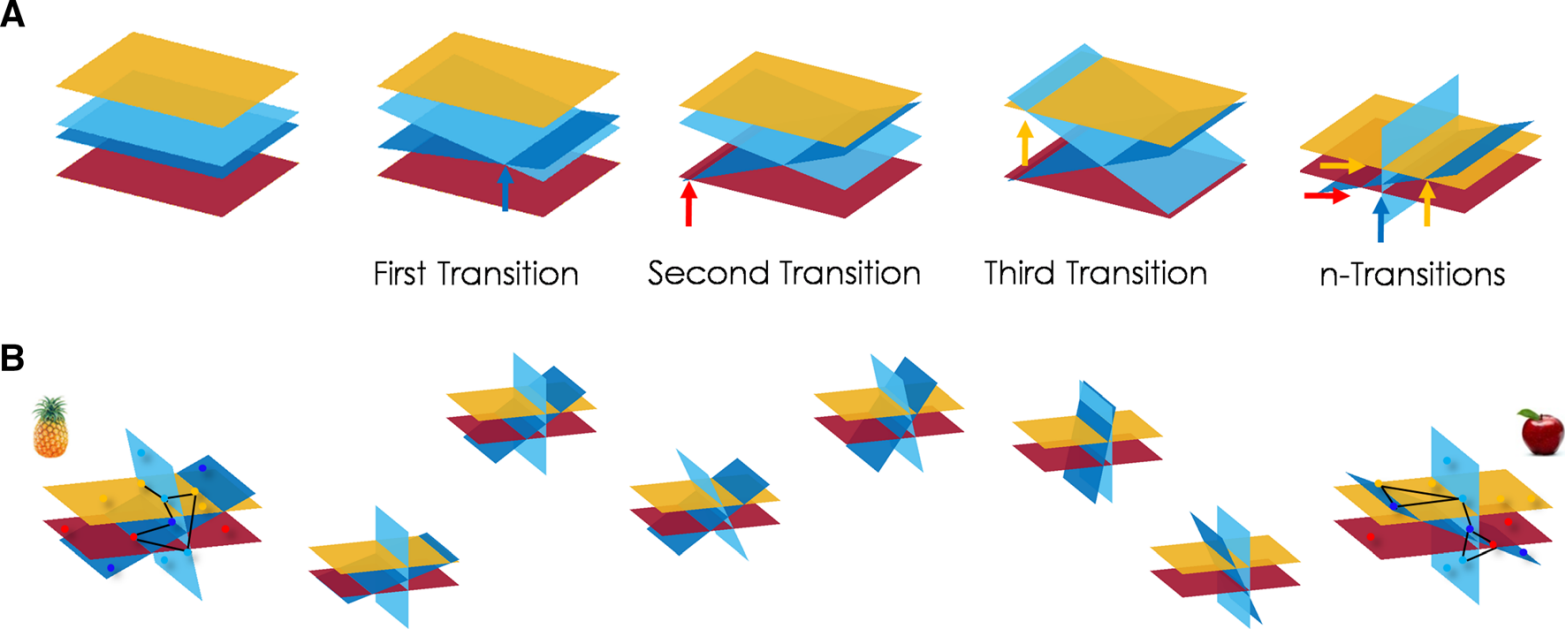
Consciousness interaction and phenomenology. **a** Different biobranes and autobranes compositional interactions generates different transitions and new local-global systems. First transitions may correspond to wakefulness, followed by transitions representing phenomenal experience, awareness, and other transitions related to more complex phenomenological experience, access consciousness and self-reference. **b** Different dynamical biobranes configurations, number and types of biobranes involved, degrees of interaction, regions of an intersection, types of oscillation, among others, would correspond to the phenomenology of conscious experience. Here, an example of autobranes as network configurations from the content of pineapple to apple, and their dynamical changes. The in-between configurations represent the dynamical evolution of these layers: the blue layers change the position while the others remain fixed. These changes inform about other states

The multibrane organization for living systems is called into play with the main motivation of unifying phenomenal and access consciousness in one single structure. For example, interactive biobranes and autobranes create new topological paths trough inter-interactions (Fig. 4a), generating dynamical phase transitions (Werner 2012, 2013). Then, the global unconscious experience defined in humans may relate to the first configuration of non-interacting autobranes, followed by a first transition where two or more autobranes start to interact. As soon as more biobranes and autobranes get involved, a second transition is defined, until all possible biobranes and autobranes under interaction form a global and consistent topological new multistructure (Fig. 4a). These transitions are triggered by biobranes interactions and co-arise with phenomenal and access aspects of experience.

These biobranes dynamically evolve and interact, like two overlapping amoebas or cellular membranes. Some of these interactions trigger transitions that may correspond to relevant biological processes for the living organism. Others may correspond to aspects of consciousness such as wakefulness, phenomenal consciousness, subjectivity, access consciousness (i.e. knowing about the content of experience) and metacognition (i.e. the capacity to inform about the processes on the contents of experience, knowing that I know). Once these reciprocal actions have emerged, each biobrane monitors the others without any biological dominance among them. It implies that if one process disappears, the awareness associated with that process in the whole system also disappears.

### Phenomenology of consciousness

Following the living metaphor, biobranes care about its processing as part of its biological requirements. Since biobranes are living structure, their interactions may express the dispositions and preferences of the whole agent (Cleeremans 2011), like any animal interaction exhibits their dispositions and preferences. To satisfy their biological requirements, biobranes care about some states more than others, such as one cares about some temperatures that correspond to original survival living preferences. These preferences result from learned biobrane interactions and the respective co-emerging experience, such that each biobrane also cares about the processing of those other biobranes which may directly affect them. It is analogue to any living being acting and reacting to different stimuli and contexts to ensure its survival. The whole system and its parts try to balance out their coupling and decoupling, as part of their biological demands.

It generates a notion of phenomenal experience, expressed in the next proposition:

#### Proposition 4

*The types of biobranes, autobranes and the degree to which they interact with each other co-arise with the structure and content of experience.*

In other words, depending on the (i) degree and types of interactions, (ii) number and types of interacting biobranes, (iii) dynamic zones of an intersection, and (iv) oscillatory mechanisms involved, a certain experience co-arises (Fig. 4b): the whole system feels one or another feeling, the experience evolves in one or another form, the content is about one or another element. This experience is not, however, unidirectionally dependent on these biobranes interactions, but both, experience and biobranes co-determine each other. The structure and content of experiences depend on the degree, types, etc. of biobranes and autobrane interactions, but also the structure and content of experience affect the degree, types, etc. of the interactions on which they depend.

Other approaches, such as harmonic modes to conscious states (Atasoy et al. 2017), also suggest that oscillations giving by their harmonic structure are related to specific phenomenal experiences, but this approach ends up reducing that experience to brain interactions only, instead of emphasising its co-dependence character. In our case, emergent properties are as important as sub-emergent properties of systems (“downward causation”). In other words, different oscillatory modes may correlate with phenomenal experience, e.g. resonance between different branes may involve access consciousness, while dissonance, lack of that access, or even further, representing different moods of the conscious animal. However, experience also triggers those oscillations in a co-determined balancing of branes interactions and experiences, creating a new form of oscillatory plasticity where sub-emergent properties play a crucial role (Rodríguez 2008).

## Implications and predictions

In our closed biobranes composition framework (CBC), conscious experience is understood as a process which mainly interferes with internal brane integration in favour of global flux of activities and influences among biobranes. First, the activity of independent molecular, cellular, glia, and neural biobranes would correlate with an *unconscious* stage. Second, the conscious stage co-arises with the activity of now compositional interacting biobranes as the non-trivial composition of a new whole system. Both systems as a composition of closed biobranes form a new operationally closed whole multibrane. Therefore, conscious processes are related to the closed activity supported in particular by cellular self-generated activities (Llinás and Paré 1991; Llinás 2003) (in autobranes) and in general by biological autonomous systems (in biobranes).

### Experimental implications

**Fig. 5:**
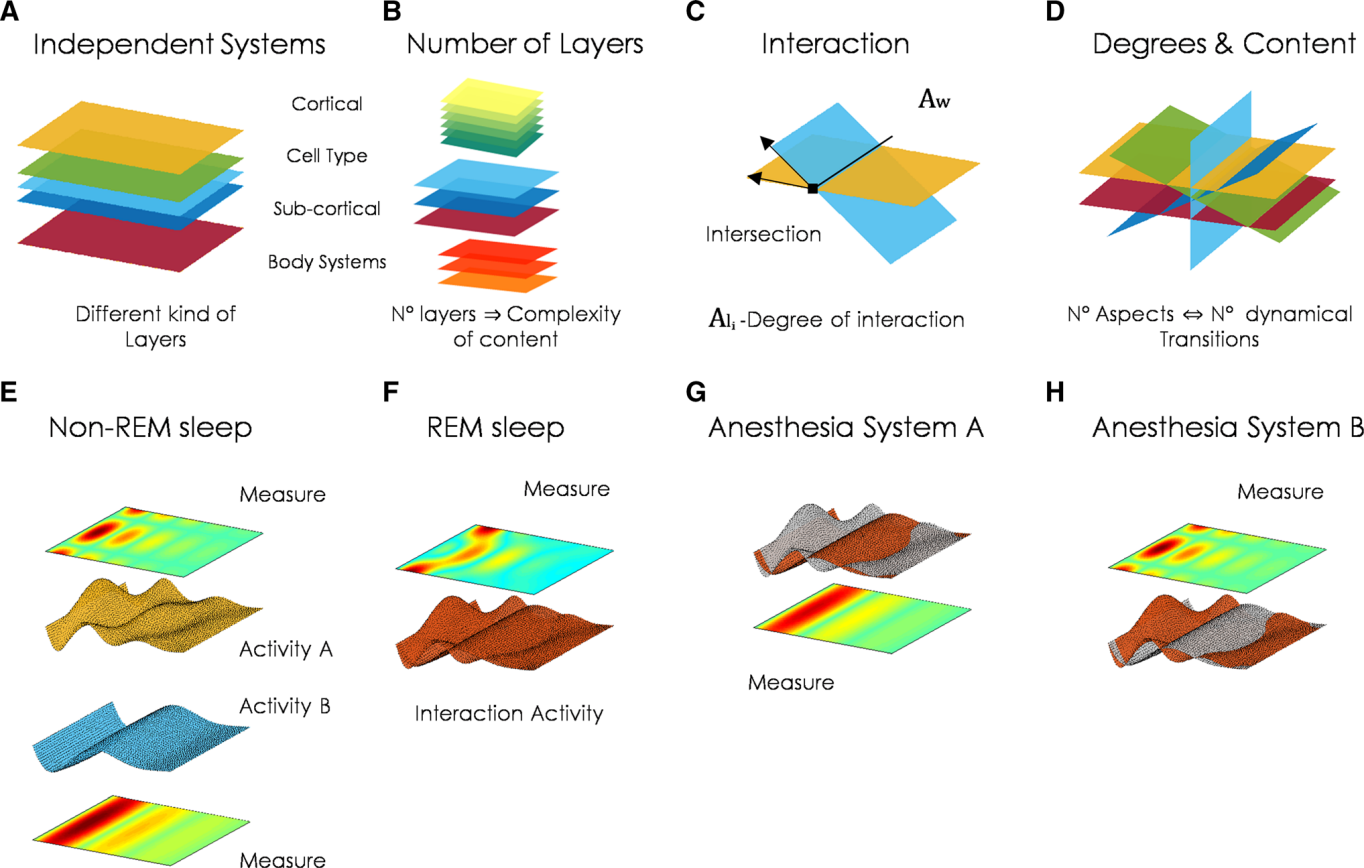
Implications and predictions. **a** The model predicts a unique multibrane division. In addition to neural assemblies, this division also includes other cell types and more general body systems. **b** More biobranes imply richer and detailed content of experience. **c** Interaction between biobranes as a multidimensional measure of consciousness (local and global biobrane autonomy) in the context of disorders of consciousness, anaesthesia protocols, sleep and psychedelics. **d** Spatial-temporal interactions (place, biobranes involved) and mechanisms (resonance, superposition, among others) may account for the details of phenomenological experience and distinctive/unique subjective conscious experiences. **e** A two-brane-system configuration in non-REM sleep. **f** A two-brane-system configuration, interacting during REM sleep. **g** A two-brane-system configuration, A and B, anaesthesia affects only one of the systems (system A). **h** A two-brane-system configuration, A and B, anaesthesia affects the other system (system B). **e**,**f**,**g** and **h** are toy examples

The model predicts the uniqueness of the multibrane division (Fig. 5a). A mathematical approximation of autobranes is the use of layers in a multilayer network (Signorelli et al. 2021). The conditions of coupling and splitting layers mathematically require a unique set of layers (see Signorelli and Joaquin Diaz 2021 for details). This layer division implies a unique criterion, in the context of conscious experience, to parcel the brain and body in terms of anatomical, metabolic, and functional biological membranes. Eventually, this approach may overcome limitations of current brain divisions (Arslan et al. 2018). Some of the autobranes/layers may represent the dynamical activity of cortical layers, sub-cortical regions, different types of cell assembly, and probably also molecular gradients acting as autonomous systems.

Identify these autobranes is not easy, but it is possible. The task requires the isolation of different brain regions, different types of cells and brain-body systems considering anatomical, metabolic (energetic exchange), and functional aspects. One empirical approximation is functionally isolating regions using anaesthetics. For example, recent evidence identify the cortical pyramidal cells in layers V and its modulation of brains states associated with consciousness (see Suzuki and Larkum 2020). Another alternative is to approximate autobranes with brain-waves incorporating body interactions. In this case, we need to isolate brain-waves and identified them with fix brain regions during deep sleep, like harmonic brain modes (Atasoy et al. 2016), but with further functional restrictions. Then, we can measure their intrinsic changes, couplings and splittings during awake and other conditions. A simpler functional approximation is to identify the intrinsic oscillation of brain regions during deep sleep together with their anatomical connections, using techniques of multilayer dynamical models (Cabral et al. 2017). Then, we can group similar inner frequencies in layers and study their evolution across other conditions. More detailed methods may target other aspects of autobranes, and new equipment such as deep optical modulation (e.g. fast high-resolution two-photon microscopy (Zong et al. 2017)), higher fMRI resolution (e.g. 11.7 Teslas, Nowogrodzki 2018) and layer-fMRI analyses (Huber et al. 2020) may help to find and define these empirical autobranes and their inner activity.

Another prediction of our framework is the direct relationship between the content of experience and number of biobranes and autobranes involved (Fig. 5b). According to complex systems theory, more configurations of interactions implies more complexity. Therefore, in our model, richer or detailed perceptual experiences would involve more biobranes. Simultaneously, having more biobranes implies the possibility of richer experiences. As such, these biobranes and autobranes are restricted by, as well as restrict different aspects of the environment (Signorelli et al. 2020). Consider a hypothetical example: the human cortex is divided, by convention, into six anatomical layers. Some anomalies are related to the disruption of these layer’s configuration (LoTurco and Booker 2013) (but see (Guy and Staiger 2017)). Also, their density and structural changes among those layers are associated with a distinctive marker of human cognition (DeFelipe 2011). If some of these layers correspond to the more complex autobranes defined above (not just to the anatomical layer division), it may support the common assumption that human experience conveys richer and detailed contents in comparison with other animals that show traces of less developed cortical layers (DeFelipe 2011). A testable hypothesis would state that some of these structural and functional cortical organizations may correspond to the autonomous biobranes defined by our conceptual model.

The model may also inspire new measures of consciousness as the degree of biological autonomy on each biobrane $A_{l}$ and the whole multibrane system $A_{w}$ (Fig. 5c). Living systems produce more of their complexity compared with what is produced by their environment. Biobrane autonomy $A_{l_i}$, with $i={[1,... n]}$, *n* number of biobranes, is then defined as the complexity of the biobrane $C_{l_i}$ divided by the complexity of the environment $C_{w}$, that corresponds to the union of other biobranes forming the whole system. For that measure, a value greater than 1 would mean more autonomy (Fernández et al. 2014). Similarly, the whole system autonomy is defined as $A_{w}=C_{w}/C_{E}$, where $C_{E}$ is the complexity produced by the environment outside the biobranes union. These definitions assume a generalized version of autopoiesis and autonomy (operational closure) based on notions of information that allow us to consider systems that self-produce its organization instead of their components. Moreover, complexity is defined as $C_{j}=E_{j}*S_{j}$, where $j={[l, w]}$. $E_{j}$ represents emergent properties as a group of new chaotic patterns in the system, i.e. new properties of a system which are not present in their elements. $S_{j}$ is the system self-organization, in the form of organized patterns that appear from local patterns interacting (López-Ruiz et al. 1995; Fernández et al. 2014; Gershenson 2015). Therefore, a high value of complexity requires a balance between emergence (chaos) and self-organization (order) (López-Ruiz et al. 1995). This consequence is equivalent to the required balance of integration and segregation/differentiation in the early version of integrated information theory (IIT). However, in our model, complexity by itself is not enough to define the conscious capabilities, as well as these interactions may correspond to more general body interactions and not only neural events.

Consciousness would require biological autonomy, specifically a decrease of $A_{l}$ while increase in $A_{w}$. This recovers the observed increase of complexity values on the whole system as a consequence of decreasing the biobrane autonomy $C_{w}=C_{l}/A_{l}$. Accordingly, $A_{w}$ values increase as the biobranes start to interact and compound the multibrane structure. The usual interpretation in the form of correlated activity brings the prediction that interconnectivity between autobranes increases with awareness, i.e. biobranes become coupled and having different types of influences among their dynamics: correlations between autobranes and their nodes increase. On the contrary, inside autobranes, intraconnectivity would decrease when awareness increases. Network analyses in physiological data and brain-body coupling systems seem to support these predictions (Bashan et al. 2012; Stankovski et al. 2016). Taking these biobranes and autobranes as independent living systems that interact to provide and obtain biological resources, this conclusion is not surprising. During their interactions, both systems depend on each other (signifying conscious processes). After these interactions take place, they need to come back to their intrinsic independent non-interacting activity, and therefore recovering their autonomy (signifying sleep states).

Measures of autonomy and the multibrane/multilayer structure also support multidimensional measurements of consciousness (Bayne et al. 2016). For example, differences between sleep stages and anaesthetics transitions would end into different values of that autonomy levels, among the different biobranes that are being affected. During deep sleep, it may be that biobranes and autobranes are naturally disconnected (Fig. 5e) or the connection has a different structure. During REM, some biobranes become disconnected, but others may still interact (Fig. 5f). In the case of different anaesthetics, some of them may act in part of the system’s branes but not in others; generating various forms of splitting biobranes (Fig. 5g, h). During non-conscious conditions, such as sleep stages and different anaesthetic, one biobrane may drive the interaction, breaking the balance requirement and reducing values of $A_{w}$. For instance, anaesthetized patients will present low values of $A_{w}$, while awake subjects will have maximal values according to a fix and normalized scale. Moreover, the sudden emergence in elderly subjects is linked here with an intrinsic change on the autobranes orientation that impacts on the functional distance between biobranes. It makes the biobranes suddenly interact once the effect of anaesthetics starts to decay, avoiding the usually smooth time transition. Asymmetries between anaesthetics induction and emergence (hysteresis) are explained by their actions on one or another biobrane with different intrinsic dynamical properties, that impact differently on the rest of the biobranes and trigger dynamical transitions in different order. Then, the global recovery is influenced by this biobrane recovery, while the others remain intact. Therefore, the coupling and splitting of that biobrane generate differences in the time and concentration effect of anaesthetics. So the type of brane, orientation and system disrupted play a crucial role in the model, and local and global autonomy values, as degrees of interaction between different biobranes, would form a global multidimensional consciousness quantifier (Bayne et al. 2016).

As a consequence, a breakdown of these biobranes and interacting structure implies a disruption of the usual conscious experience. Due to this organization, some sections of the biobranes may participate in more intersections than other regions. Therefore, their intrinsic functional activity being notoriously disrupted under conditions of global rearrangement such as chronic disorders of consciousness and/or sleep (Signorelli et al. 2021). It may generate the impression that some areas define a hierarchy of active regions or hubs, such as proposed by global neural workspace theory (GNW). Once biobranes naturally decouple, these hierarchies would appear as disrupted, but in reality, they go back to their intrinsic dynamic. For multibranes, it means that any dynamic impairment of a biobranes will lead to the awareness associated with that biobranes disappear. This impairment may also affect the global biobranes balance through decoupling some biobranes. Therefore, the local causal driven forces that generate similar global disruptions would correspond to disruptions on different biobranes. These disruptions become a common mechanism for loss of consciousness, at the same time that they save the specificity of different impairments. In other words, independently of the molecular pathways of different anaesthetics, stages of sleep or localization/types of brain injuries, the dynamical disruption correspond to a re-arrangement of biobrane organization distant from the awake condition. In summary, a breakdown of seemly hierarchical organization indeed corresponds to break of natural balance from a conscious interacting multibrane structure to an unconscious partially non-interacting structure

### Theoretical implications

The multibrane framework forces us to specify: i) the type of organizational structure (types of networks, multinetwork, membranes, etc), ii) the components of these structures, iii) the interactions among components, and iv) the explicit mechanism supporting conscious experience^4^. This situates our model within the discourse of current models of consciousness. For instance, the commonalities and differences with IIT and GNW are summarized in Table 3.

**Table 3:** Comparison of three models of consciousness according to a multilevel interacting framework

**Model**	Structure	Components	Interaction	Consciousness
IIT	Monoplex-time evolving Net	Physical systems	Cause-effect interactions	Maximal causal integration
GNW	Multilevel-time evolving net	Neurons	Inter-area action potentials	Broadcasting from a global workspace
CBC	Dynamical multibrane	Biological autonomous systems	Multiple interactions	Closed composition of biobranes

For example, the GNW and IIT are neither embodied nor compositional models, since they reduce consciousness to only properties of neural interactions. On the contrary, our discussion proposes that the relevant interactions to conscious experience are found on the multiple levels of the organizationally closed biological system. It entails multiple types of interactions. Contrary, GNW emphasises the role of broadcasting electrical activity from certain areas of the brain to other areas (Dehaene and Changeux 2005; Van Vugt et al. 2018), and IIT focuses on intricate mechanisms of causal integration at the level of neural assemblies (Oizumi et al. 2014). A multibrane extended framework subsumes the network structure of these two models: while GNW seems more general than IIT, our model can be seen as an extension of GNW but avoiding functionalism and philosophical reductionism. Recently, new dynamical approaches also suggest that other new physical principles are playing a relevant role in the conscious activity. Among them are harmonic modes (Atasoy et al. 2017) and criticality (Werner 2013; Tagliazucchi 2017). Following our general framework, we believe the concepts introduced here may integrate all these principles while emphasising the importance of formal biological definitions in models of consciousness (Signorelli et al. 2020). This is a crucial step forward to define a formal model of consciousness, namely, models which make explicit their theoretical and experimental assumptions (Kleiner 2019).

### Philosophical implications

Our approach also has philosophical implications which make it a promising model against other scientific models of consciousness. First, it is a *nondualistic framework*. Second, it acknowledges the *primacy of embodiment*. Third, it acknowledges the *primacy of consciousness*. Fourth, it is *pragmatic*. In this subsection we briefly discuss why our framework is a relevant conceptual apparatus.

#### The nondualist framework

Our model starts from a *radical embodiment reformulation* of the mind-body problem (Thompson 2007, 2014). The mind-body problem arises when theories of consciousness assume the primacy of substance-like ontological objectivity, i.e. elements having a uniform and constant properties. Therefore, the focus is on those physical parts, cells, neurons, regions of the brain, from which the experience is thought to emerge as a whole (Searle 2000). However, the existence of subjective ontologies seems not reducible to objective ones (e.g. the redness of the red is not yet explained by the light wavelength or any elaborated neural event). Then, the concept of qualia is coined to extend the same uniform and constant substance ontology, but this time for subjectivity. This strategy leads us to irreconcilable mind (subject) and matter (object) ontologies. Rather, our model makes use of process-ontologies [25], i.e. what exists are processes of transformations (as opposed to constant substances). These ontologies seem better suited to explain the idea of the living body and the lived body (consciousness) as two modes of appearance of one and the same body: mind and body are not separate. Mind and body are different modes of the same process: closure. This closure leads us to the property that makes living differently than nonliving and consciousness different than unconsciousness. In consequence, any mathematical or computational structure/architecture supporting consciousness should constitute and realize consciousness, as well as its components, constitute and realize living being.

#### The primacy of embodiment

Our model builds on the idea of *primacy of embodiment*, implying two important aspects. First, we proposed that a sound and scientific model of consciousness must build on a principled definition of the living body. Current models treat biological processes (autonomy) as mere physical processes (heteronomy). Rather, in our model, biological processes always involve organizational closure, with all its implications for a proper paradigm of biological interactions (Maturana 2011; Maturana and Varela 1998). Second, our framework builds on the notion of *embodiment* of neural activity. Current models reduce consciousness to brain states. Rather, in our approach, consciousness is related to multidimensional biological processes which go beyond mere neural events. Via the mathematical machinery for biobranes and autobrane, our model may allow future mathematical precise descriptions of biological autonomous processes across various types of cells of the living system.

This embodiment has strong implications for artificial life and consciousness. Materialistic approaches imply that replicating the *computational architecture* of neural systems convey artificial consciousness (Dehaene et al. 2017; Tononi and Koch 2015). We reject this conclusion unless the unique biological features of biological autonomous systems and the organizational multibrane structure are primarily replicated. If the biological autonomy is replicated as an essential prerequisite to achieve consciousness, life needs to be replicated as requisite of biological autonomy (Signorelli 2018a, b). In this context, a thermostat is “less conscious” than a rat not because of its information capacity, or because it misses all the relevant functional architecture and electrochemical mechanisms of a neural workspace. The thermostats or any non-living being is unable of awareness just because they are non-living systems and as such, they lack biological autonomy. At the end of the day, only living beings seem to have conscious experiences, hence the living organization is indeed important.

#### The primacy of consciousness

Our model builds on the idea of *primacy of consciousness*. There are two important aspects. First, consciousness is ineliminable and cannot be reductively explained in terms of brain states or even the living body. While current nondualist models reduce consciousness to electrochemical processes, our approach does not. Rather, biological processes and consciousness dynamically co-arise. Therefore, they are co-dependent: Consciousness depends on biological processes, but it also affects the biological processes on which it depends. Second, many interpretations of the primacy of consciousness subscribe to dualism or panpsychism. Our model does not. Dualism says that “the physical” and “the mental” have distinct natures. Panpsychism says that every physical phenomenon, intrinsically, carries some measure of consciousness. However, our model is neither dualistic nor panpsychism. Rather, it builds on a non-dualistic framework in which physical being and experiential being imply each other (Thompson 2014). Mind and body are (i) neither separate, (ii) nor only mind, (iii) nor only body. Our approach is a non-dualistic framework that acknowledges the simultaneous primacy of embodiment and consciousnes.

#### Pragmatic usefulness

The philosophical attitude supporting our model is strongly influenced by the modern approach to formal mathematics after Gödel’s incompleteness theorems (Gödel 1931). It means that any set of axioms is a useful set of axioms in the context in which formal theories are based on, but the same set of axioms may not be useful in another theoretical contexts (Gershenson 2013). It does not mean that these axioms are true or false, as well as Newton’s laws are not true or false because they work at some scale but not at others (e.g. macro-scale and micro-scale). On the contrary, neuroscientific models of consciousness assume that something true, objective and invariable is said about consciousness and its mechanisms. However, the operation of observing distinguishes between what we, as observers, can say about any system that appears to us from what we say about what may occur in the internal operation of the observed system (Maturana 2011). There is nothing obscure or restricted to the micro or atomic world, we only observe transformations, and it becomes essential to any introspective operation of observation: a feature which is not objectively measurable. As pointed out by Box, “all models are wrong” (Box 1976), and as emphasized later, “but some are useful”. Hence, models are descriptions of the modelled phenomenon, and as such, models depend on the observer (Gershenson 2013; Maturana 2011).

Therefore, we just intend to introduce a useful framework guiding further experimental hypotheses through useful axioms and definitions (Signorelli and Joaquin Diaz 2021). Different than other models, our framework addresses the complexity of the conscious phenomenon, not through reducing the system to a certain group of ontological laws or components, but composing abstractions and proving its power explaining and predicting new features of the phenomenon in question. In other words, our model is a pragmatic phenomenological approach, not an ontological one. It makes our conceptual apparatus a new promising approach to the biology of conscious experience.

## Conclusions and further work

Across these pages, we introduced a conceptual apparatus to explore the biology of conscious experience. We invoke biological closure to rescue the living structure as an essential requirement of radical embodiment. Compositionality is called into play to discuss different levels of closure and its relationship to a multilevel structure in the brain-body. Biobranes and autobranes are conceptualized to model these compositional closures in the context of conscious experience. This framework centres on the living and its unique organizational structure: i) the co-dependency between the living body and the lived body, and ii) a multilevel organization to reconcile different brain-body signatures of experience. This multilevel organization accounts for the various biological processes, cell types and biological systems that relate to consciousness. As such, our approach is simple and eventually a mathematical theory inspired by enactive and embodiment approaches to conscious experience.

Moreover, our model subsumes some concepts from previous network models (Dehaene and Changeux 2011; Tononi et al. 2016), but surpasses them by making a clear distinction regarding the biological definition of the elements that can form interacting biobranes and what it means for them to be compositional. It turns to be a clear advantage over other models: Different from previous models of consciousness, our framework states co-dependencies between brain-body and experience, avoiding reductionism.

In future attempts, we expect to develop the mathematical and empirical machinery to test the main propositions and predictions. It might consider biological autonomy and closure at different levels. Operational definitions of biobranes and autobranes are a crucial step forward to implement biological autonomy as a local and global measurement of the degree of brane interactions and therefore, of multidimensional signatures of consciousness. Moreover, phenomenological approaches such as neurophenomenology (Varela 1996) and micro-phenomenology (Petitmengin et al. 2019) shall be at the centre of that testing, specifically to test the relationship between biobranes interacting and the phenomenology of conscious experience following our last proposition. We are aware that, all together, it conveys an ambitious research program.

Finally, we expect that some of the concepts introduced across these pages inspire new theoretical and empirical models of consciousness. Importantly, these concepts offer potential answers to the motivational questions at the beginning of this article: i) biobranes may define relevant brain-body regions and interactions, ii) conscious experience does not emerge, but co-arises with compositional closed interactions in a living multibrane structure, and iii) machines are not conscious unless they replicate the compositions of closure, from living to consciousness.

We believe that the only way to solve the apparent gaps between body and mind is through integrative models and therefore through new metaphors for biological neuroscience of consciousness.

## Footnotes

1 Varela in his writings does not distinguish between organizational and operational closure. He uses the two terms interchangeably.

2 A Mathematical structure is the set of mathematical objects and their relationships that satisfied certain axioms.

3 Claiming a similar mathematical structure means claim similar relationships. This theoretical hypothesis can be tested using the machinery of Category theory.

4 Mechanisms of consciousness applies for reductionist approaches. In our framework, experience and living are at the same level, therefore a reductive mechanism does not fully apply.
